# Virtual cut flow, an innovative noninvasive 4D ASL MRI biomarker of EIC bypass patency

**DOI:** 10.1007/s10143-025-03618-7

**Published:** 2025-05-27

**Authors:** Antoine Devalckeneer, Geoffrey Haddad, Gregory Kuschcinski, Xavier Leclerc, Philippe Bourgeois, Nadira Delhem, Tomas Menovsky, Rabih Aboukaïs, Martin Bretzner

**Affiliations:** 1 Neurosurgical Department, CHU Lille, Roger Salengro Hospital, Lille, F-59000 France; 2 University Lille, INSERM, U1189-ONCO-THAI-Image Assisted Laser Therapy for Oncology, Lille, OncoLille F-59000 France; 3 Neuro-Interventional Department, CHU Lille, Roger Salengro Hospital, Lille, F-59000 France; 4 Neuro-Radiology Department, CHU Lille, Roger Salengro Hospital, Lille, F-59000 France; 5 Department of Neurosurgery, Antwerpen University Hospital, Antwerpen, Belgium; 6 University Lille, INSERM, CHU Lille, U1172 - LilNCog - Lille Neuroscience & Cognition, Lille, F-59000 France

**Keywords:** Extra-intracranial bypass, Virtual cut flow index, 4D ASL

## Abstract

Extracranial-intracranial (EIC) bypasses are used to treat Moya-Moya syndrome and complex aneurysms. Evaluating bypass patency is vital. Current methods include intraoperative techniques like indocyanine angiography, micro-doppler probes, and postoperative non-invasive imaging. However, adjudicating the frequent discordant imaging results is done via conventional digital subtraction angiography (DSA) despite its associated risks. Our study aims to quantitatively evaluate bypass patency by introducing a novel biomarker, the virtual cut-flow index (VCFI) derived from 4D-PACK MRI imaging. We prospectively reviewed consecutive adult patients who underwent bypass surgery at our institution between January and October 2023. MR angiography was performed, employing 4D-PACK (four-dimensional pseudo-continuous arterial spin labeling (4D-pCASL)-based angiography using CENTRA-keyhole and view sharing) imaging. We measured arterial signal intensity using regions of interest (ROIs) placed on the superficial temporal artery (STA) and a branch of the middle cerebral artery (MCA) post-bypass. We modeled signal evolution using linear regression and extracted slope α coefficients and then calculated the virtual cut-flow index (VCFI) using the equation VCFI = αMCA/αSTA. We included 18 patients corresponding to 19 bypasses. The median VCI was significantly higher in patients with a patent bypass compared to those with an occluded bypass (87.33 vs. 19.87%; *p* < 0.05). The median αMCA coefficient was significantly higher in patients with a patent bypass compared to those with an occluded bypass (69.21 vs. 11.34; *p* < 0.05). The median αSTA coefficient was significantly higher in patients with a patent bypass compared to those with an occluded bypass (102.74 vs. 44.74; *p* < 0.05). We introduced a novel noninvasive biomarker, the virtual cut-flow index, as a new means to assess bypass patency. While DSA remains the gold standard, VCFI offers a promising avenue for quantitative evaluation, potentially enhancing postoperative monitoring and influencing surgical planning.

**Supplementary Information:**

The online version contains supplementary material available at 10.1007/s10143-025-03618-7.

## Clinical trial number

Not applicable.

**Supplementary Information:**

The online version contains supplementary material available at 10.1007/s10143-025-03618-7.

## Introduction

Extra-intracranial bypass is a primary treatment for adult patients with Moya-Moya syndrome and is also increasingly utilized for managing complex aneurysms [2, 3, 11, 19, 26, 32]. Among revascularization techniques, the superficial temporal artery bypass to middle cerebral artery (STA-MCA) is the most commonly performed [1, 2, 17, 19, 21, 26]. Ensuring patency of the anastomosis during the early post-operative window or during follow-up is essential to ensure best clinical outcome [1, 2, 45].

Accurate evaluation of the patency of the donor and recipient vessels as well as the bypass is done before, during, and after surgery [1, 2, 8, 10–13, 16, 18, 19, 22, 26]. Cerebral DSA is the gold standard assessment technique to assess temporal and MCA arteries before surgery. Those arteries are then evaluated during surgery with the Cut Flow Index (CFI), defined as the ratio between bypass flow and donor vessel cut flow [5, 8, 10, 18]. This measure has been shown to predict the success of the bypass and to correlate with long-term patency [8, 10]. Intraoperative assessment of anastomosis patency can be performed using indocyanine angiography [1, 12, 14, 16, 22, 27, 42, 44] for qualitative evaluation and micro-doppler probes [1–3, 5, 8, 10, 35] for quantitative evaluation.

During follow-up, noninvasive imaging techniques such as transcranial Doppler [1, 2], CTA [1, 2, 13], and MRI [1–3, 15, 23, 41, 43] offer valuable information into bypass patency. However, their findings should be interpreted with caution due to the influence of local anatomical, physiological, and pathological factors [1]. In case of discrepant findings, conventional digital subtracted angiography, including supra-selective catheterization of the donor vessel, is suggested [1]. However, postoperative DSA exposes the patients to some complications, particularly in the early postoperative period. Therefore, there is a pressing need for an innovative technique that can reliably and noninvasively assess bypass patency, minimizing patient risk while ensuring accurate and consistent evaluations.

The 3D arterial spin labeling (3D-ASL) serves as a non-invasive imaging technique widely used in clinical practice to evaluate intracranial perfusion using protons in arterial blood as a contrast agent [33, 36–39]. Its utility extends to non-contrast-enhanced intracranial four-dimensional MR angiography (4D-ASL MRA), facilitating dynamic flow evaluation in conditions such as Moya-Moya disease (MMD) [1, 36] and arteriovenous malformation. Recent advancements in 4D MRA, leveraging pseudo-continuous ASL (pCASL) combined with contrast-enhanced timing-robust angiography (CENTRA) k-space sampling with the keyhole and view-sharing (4D-PACK), have shown promise in qualitatively assessing extracranial-intracranial (EIC) bypass patency [36, 38, 39]. Yet, a quantitative analysis of bypass patency remains unexplored.

Our study aims to address this gap by quantitatively evaluating bypass patency trough the introduction of a novel biomarker, the virtual cut-flow index (VCFI) derived from 4D-PACK MRI imaging.

## Methods

### Population

Our study prospectively included consecutive adult patients (age > 18 years) who benefited from bypass surgery and follow-up at our institution between January and October, 2023. All patients with Moya-Moya syndrome/disease and complex aneurysmal diagnosis were addressed to our institution for consultation and advice allowing exhaustive and consecutive enrollment.

## Institutional multidisciplinary management in bypass surgical approach

Each case is discussed collegially during neurovascular meetings involving neurologists, interventional neuroradiologists, and neurosurgeons.

Concerning *complex aneurysm*, we used “high flow bypasses” (carotid–sylvian anastomosis; > 60 mL/min) and “low flow bypasses” (STA-MCA anastomosis). In our experience, intraoperative analysis using the Cut-Flow Index has shown that the term ”low-flow” bypass may be inappropriate, as flow rates exceeding 50 mL/min have been observed in STA–MCA anastomoses. This intraoperative micro-Doppler measurement of the Cut Flow Index is directly correlated with the long-term patency of the bypass. This observation has led us to advocate for an STA–MCA bypass in first line, which we prefer to describe as a “protective bypass”, prior to performing a high-flow bypass – or even as a substitute. Another key element in the decision is the evaluation of distal collateral circulation, which may be indirectly assessed during surgery by applying the Clip Occlusion Test. This enables both qualitative haemodynamic evaluation via fluoroscopic angiography and quantitative assessment via micro-Doppler following exclusion of distal perfusion. Such assessment allows us to refine the choice of bypass strategy, tailoring it to the patient’s individual vascular anatomy and needs.

Concerning *Moya-Moya*, all patients undergo optimization of medical management and are prescribed lifelong aspirin therapy without interruption with exception in case of hemorrhagic stroke. Following this, they are scheduled for Transcranial US-doppler, CTA, outpatient cerebral angiography to assess Suzuki staging and evaluate cerebrovascular reserve using both 99mTc-HMPAO SPECT with Acetazolamide and cerebral perfusion MRI. In patients with impaired cerebrovascular reserve or those presenting with symptoms, surgical intervention is indicated. For adults, we prioritise direct cerebral revascularisation via STA-MCA M4 bypass, favouring a single-donor included in this study/double-recipient technique when feasible. This is combined with local indirect revascularisation within the scope of encephalo-duro-myo-synangiosis to maximise outcomes.

## Multimodal bypass patency assessment necessity [1]

During study period, 12 patients undergo follow-up imaging within 72 h, including cerebral CT angiography, 4D ASL MRI, and cerebral angiography (DSA). At six months, a comprehensive evaluation is performed using cerebral perfusion MRI and 99mTc-HMPAO SPECT with Acetazolamide in cases of Moya-Moya to monitor the effectiveness of the intervention and guide further management. All patients are followed up annually with clinical evaluations, including at least one invasive (DSA) and systematic non-invasive imaging (CTA, MRA, and transcranial Doppler). Six patients who have previously undergone surgery benefit from 4D ASL MRI in parallel with their scheduled exams.

### Ethics

The “Lille Ethics Committee” and the “Collège de Neurochirurgie” approved the study (n° IRB00011687 College de neurochirurgie IRB #1: 2024/32). Informed consent was obtained from all participants if conscient before surgery and from related family in case of comatose patient.

### MR angiography

The MR scan was performed on Achieva 3.0T dstream system (Philips Healthcare) using a 32-channel head coil. The parameters of 4D-PACK were as follows: 3D T1 turbo field echo (TFE) acquisition; repetition time/echo time (TR/TE) = 6/1.96 ms; field of view (FOV) = 200 × 200 × 120 mm^3^; flip angle = 11°; resolution = 1 × 1.3 × 1.6 mm^3^; keyhole percentage: 75%; 6 time points with different label durations = 100, 400, 800 1600, 2200, 2800 ms; and post labeling delay = 50 ms. The Compressed SENSE technique (C-SENSE) with a factor of 6.5 was used to accelerate the sequence (ref), allowing for a scan time of 5 min 5 s. Conventional techniques were performed as well: 3D-TOF, CE MRA, diffusion, FLAIR, and T2*.

### MR image analysis

**Fig. 1:**
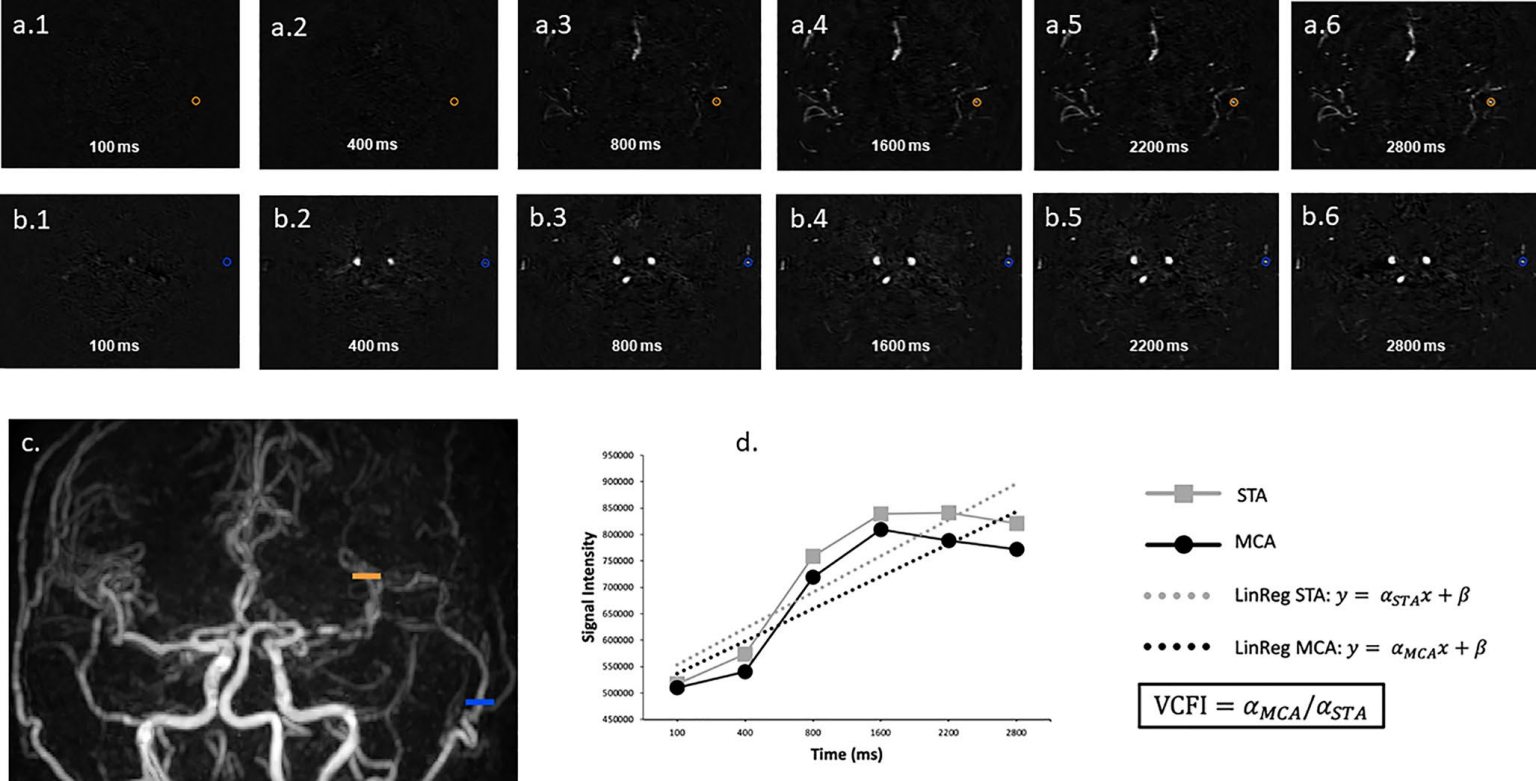
Virtual cut-flow index measurement. a.1 to a.6: axial slices at the M2 branches level as derived from image c. at different timeline t1 = 100ms, t2 = 400 ms, t3 = 800 ms, t4 = 1600 ms, t5 = 2200 ms, t6 = 2800 ms. b.1 to b.6: axial slices at the STA level as derived from image **c**. during same timeline t1 = 100ms, t2 = 400 ms, t3 = 800 ms, t4 = 1600 ms, t5 = 2200 ms, t6 = 2800 ms. **c**.: coronal MIP view showing a patent left STA-MCA bypass with ROI selection at the M2 level (orange) and STA level (blue). **d**.: signal intensity evolution over time and linear regression extrapolation where α represent the slope coefficient respectfully from MCA and STA; Virtual Cut Flow Index (VCFI) is defined as the ratio of αMCA and αSTA

Arterial signal intensity was measured using regions of interest (ROIs). Two ROIs were placed on the last time point when the vascular structures are the most visible for each bypass: one on the proximal part of the STA, the other on a M2-M4 branch of the MCA beyond the anastomosis (Fig. 1). The placement of ROIs followed a consensus reached between a neuroradiologist and a neurosurgeon, ensuring the accuracy and relevance of the measurement locations. The last time point was used as a vascular map. The vessels’ signal was then evaluated at each time point, resulting in 2 sets of 6 measurements per bypass.

### Virtual cut flow index concept

Intraoperative assessment of bypass viability utilizes the cut-flow index, which is the ratio of the post-anastomosis blood flow within the donor artery in relation to the free-flowing (pre-anastomosis) donor blood flow after it has been cut at the tip [8, 10].The free flow of the donor vessel is considered the maximum potential flow deliverable by the anastomosis. Indeed, post-bypass, a downstream resistance can be detected intraoperatively using fluorescein angiography [1, 11, 14, 16, 34, 42, 44] and postoperatively via arteriography. We have adapted the cut-flow index concept to 4D-ASL by developing a method to model intracranial flow through the analysis of arterial signal evolution upstream and downstream of the bypass. Focusing on the early phase of the Arterial Input Function (AIF), we assumed that the donor vessel’s flow could be estimated via linear regression using the equation *y = αx + β*, where α represents the slope coefficient and β the intercept. We hypothesized that the donor vessel’s slope coefficient (αSTA) indicates the maximum flow deliverable by the anastomosis, and that of the vessel downstream of the bypass (αMCA) represents the bypass flow. Thus, we modeled bypass performance by creating a metric, the virtual cut-flow index (VCFI), calculated using the equation *VCFI = αMCA/ αSTA* (Fig. 1).

### Statistical analysis

Data was analyzed using Mann-Whitney test in Prism 10.0.2 software (Graph Pad Software Inc., San Diego, CA, USA). All quoted p-values are two-sided, with *p* ≤ 0.05 (*) being considered statistically significant.

## Results

### Population

Our study included 21 patients (female to male ratio 16:5). Demographics, clinical and radiographic features are summarized in Table 1. Three patients have been excluded from analysis due to non-interpretable MRI signal related to clip artefact (1 in Moya-Moya group and 2 in Complex aneurysm group). Therefore, 18 patients corresponding to 19 bypasses have been included. All included patients presented with previous DSA, CTA and TCD confirming the bypass patency.

**Table 1:** Demographics, clinical and radiographic features

Indications	Moya-Moya (*n* = 11)	Complex Aneurysm (*n* = 10)
Graft	STA (*n* = 11)	STA (*n* = 9) Other (4)
Bypass	Bilateral (*n* = 2) Double (*n* = 3)	Double (*n* = 2)
Territory	MCA (*n* = 11)	MCA (*n* = 8) PICA (*n* = 2)
DSA Patency	15/16	12/13
CTA Patency	15/16	12/13
TCD Patency	15/16	11/13
ASL Patency	14/15	10/11
Exclusion MRI analysis	1	2 (PICA)
Age	21 Yo	44 Yo
Sex ratio F/M	7/4	9/1
Post-operative stroke	1 lacunar	2 M2 branches
mRS > 2	0	3
Mortality	0	0

STA: superficial temporal artery, DSA: Digital Substracted Angiograpgy, CTA: Computed Tomography Angiograpgy, TCD: Transcranial-Doppler, Yo: year old, F: Female, M: Male, mRS: modified Rankin Score

### Virtual cut-flow index

Native MR on axial at each time point (100, 400, 800, 1600, 2200, 2800 ms) and Maximum Intensity Projection on coronal of 4D-PACK images. Circular ROIs were placed on axial plane in straight segment of the STA (blue) and the M2-M4 MCA post-bypass branch (orange). ROIs were placed at each time point, using the last time point as reference, when maximal signal was reached in the arteries. Graph showing the evolution of the signal in the STA and the MCA, as well as the resulting linear regression line: y = αx + β. Virtual cut-flow index (VCFI) is calculated using the equation VCFI = αMCA/ αSTA. Our results are illustrated in Fig. 1.

As demonstrated in Supplementary Fig. 2., the median VCFI was significantly higher in patients with a patent bypass (87.33%) compared to those with an occluded bypass (19.87%; *p* < 0.05). The median αMCA coefficient was significantly higher in patients with a patent bypass (69.21) compared to those with an occluded bypass (11.34; *p* < 0.05). The median αSTA coefficient was significantly higher in patients with a patent bypass (102.74) compared to those with an occluded bypass (44.74; *p* < 0.05).

## Discussion

This study introduces the virtual cut-flow index (VCFI) as a novel biomarker for quantitatively evaluating extracranial-intracranial (EIC) bypass patency, utilizing 4D-PACK MRI imaging. Our findings demonstrate the potential of VCFI, showing significant differences in patients with patent versus occluded bypasses, supported by differences in the αMCA and αSTA coefficients. These results suggest that VCFI could serve as a tool for assessing bypass patency, offering a non-invasive alternative to traditional methods such as Digital Subtraction Angiography (DSA).

In steno-occlusive disorders, arterial narrowing can lead to prolonged transit delays distal to the stenosis, resulting in artefacts when employing single-PLD ASL [39]. Since the degree of stenosis varies with both disease severity and aetiology, selecting a single longer PLD may not suffice, making multi-PLD ASL a more appropriate strategy in such cases [20]. The application of 4D-PACK imaging in evaluating bypass patency aligns with the growing demand for non-invasive diagnostic tools in neurovascular diseases. Previous studies have primarily focused on qualitative assessments of bypass patency using 3D-TOF [1, 2, 24, 26, 31, 32, 43] and more recently vessel-selective 4D MRA [6, 36–39]. However, the quantitative approach provided by VCFI introduces a new dimension to bypass evaluation, offering insights into the hemodynamic changes post-bypass surgery. This non-invasive method stands in contrast of DSA, which carries inherent risks including radiation exposure, contrast-induced nephrotoxicity, and procedural complications.

Post-operatively, the technical success of the bypass can be judged by angiographic patency in addition to quantitative measurements of blood flow through the graft using PCMRA [9, 10, 25, 29, 46]. Indeed, the technique of blood flow quantification by PCMRA has been described previously. The technique has been implemented and enhanced in commercially available software called the Noninvasive Optimal Vessel Analysis (NOVA) system (VasSol, Inc., Chicago, IL).

While TOF MRA provides a qualitative assessment of the dichotomized patency of the anastomosis, 4D-pcASL allows for the quantitative evaluation of the anastomosis by delivering a robust patient-independent biomarker. Indeed, direct measurement of MRI on TOF MRA or single PLD ASL can’t be compared between patients, resulting in abstract values. By calculating the VCFI, we obtain values that can be used to find patient-independent thresholds and cutoff values. Moreover, our method paves the way for the longitudinal assessment of the STA-MCA anastomosis function across all MRI assessments. It could help detect early signs of dysfunction by studying its functional trajectory over time and might thus warrant further exploration or even reintervention before clinical manifestation. All in all, 4D-pcASL has the potential to detect preliminary signs of occlusion, whereas TOF MRA or single PLD asl can only detect anastomotic dysfunction after its complete occlusion.

The adoption of VCFI could improve the postoperative monitoring of bypass patients, enabling early detection of bypass failure and facilitating timely interventions. Furthermore, the ability to quantify blood flow dynamics through the bypass and distal vessels may provide a deeper understanding of the physiological changes following EIC bypass surgery, potentially influencing surgical planning and postoperative care [1, 2, 8, 10, 11, 25, 30, 34–37, 46].

Based on our results and the CFI principle, we proposed that VCFI > 0.5 could be interpreted as a long-term patent bypass and VCI < 0.5 as deficient or occluded bypass. In our series, the patency bypass rate is 100% if the VCFI is > 0.5. In future study, we hope our method will be validated analyzing pre-operative and post-operative patients. In this case, not only the VCFI will be validated as follow-up biomarker but also could be integrated with ultrasound micro-doppler per-operative values and merged with data originating from ^99^mTc-HMPAO [2, 4] or PET using H_2_^15^O, ^15^O_2_ or C_15_O_2_ as tracers [2, 4, 28]. Clinical and research studies, particularly in patients with moyamoya disease and intracranial atherosclerosis, have demonstrated that CBF measurements obtained using multi-PLD ASL exhibit greater sensitivity to haemodynamic changes following revascularisation compared to single-PLD ASL [20, 40, 47]. Moreover, ATT estimates derived from multi-PLD ASL have shown good concordance with established perfusion metrics from techniques such as dynamic susceptibility contrast MRI and ^15^O_2_ PET, while avoiding the need for contrast agents [7, 47, 48]. In this way, we could better quantify blood flow allowing brain mapping in relation with the bypass revascularization itself. At last, the application of 4D-PACK sequence in routine could be a substitute to other exams for asymptomatic patient or, when DSA couldn’t be performed due to the fragility of certain patients, necessitating the use of validated other methods.

The authors concede that the present study is constrained by several factors. The sample size was small, which may impact the generalizability of our findings, particularly due to the limited number of patients with occluded bypasses (*n* = 2). Additionally, the study design introduces potential selection biases and, importantly, there was no preoperative study of the signal evolution in the superficial temporal artery (STA), which could have provided valuable baseline data for comparison and further understanding of postoperative changes. This topic should be addressed in a second ongoing study. A limitation lies in the measurement of VCFI, which is a non-automated process requiring close collaboration between neuroradiologists and neurosurgeons for the placement of ROIs. Certain patients were excluded due to artifacts related to surgical and embolization materials, underscoring the technical limitations encountered in imaging postoperative neurovascular structures in patients with extensive surgical histories.

## Conclusions

This study presents the virtual cut-flow index (VCFI) as a new biomarker to assess extracranial-intracranial (EIC) bypass patency using 4D-PACK MR imaging. Results indicate that VCFI effectively distinguishes between patients with patent and occluded bypasses, supported by variations in the αMCA and αSTA coefficients. Caution due to sample size, this suggests VCFI’s potential as a non-invasive alternative to Digital Subtraction Angiography (DSA) for evaluating bypass patency, though DSA remains the gold standard due to its superior spatial resolution.

## Electronic supplementary material

Below is the link to the electronic supplementary material.

Supplementary Material 1

## Data Availability

No datasets were generated or analysed during the current study.

## Abbreviations

3D-ASL: 3D arterial spin labeling
CFI: Cut Flow Index
CBF: Cerebral Blood Flow
CBR: Cerebral Blood Reserve
EIC: External-Intracranial
MMD: Moya-Moya Disease
ROI: Region Of Interest
STA: Superficial Temporal Artery
VCFI: Virtual Cut Flow Index

## References

[1] Aboukais R, Menovsky T, Verbraeken B, Gautier C, Lejeune JP, Leclerc X (2021) The evaluation of intracranial bypass patency in neurosurgical practice. Neurochirurgie 67:125–131. 10.1016/j.neuchi.2020.10.001 33115607 10.1016/j.neuchi.2020.10.001

[2] Aboukais R, Verbraeken B, Leclerc X, Gautier C, Henon H, Vermandel M, Menovsky T, Lejeune JP (2019) Superficial Temporal artery-middle cerebral artery anastomosis patency correlates with cerebrovascular reserve in adult Moyamoya syndrome patients. Neurochirurgie 65:146–151. 10.1016/j.neuchi.2019.05.001 31185229 10.1016/j.neuchi.2019.05.001

[3] Aboukais R, Verbraeken B, Leclerc X, Gautier C, Vermandel M, Bricout N, Lejeune JP, Menovsky T (2019) Protective STA-MCA bypass to prevent brain ischemia during high-flow bypass surgery: case series of 10 patients. Acta Neurochir (Wien) 161:1207–1214. 10.1007/s00701-019-03906-4 31041595 10.1007/s00701-019-03906-4

[4] Acker G, Lange C, Schatka I, Pfeifer A, Czabanka MA, Vajkoczy P, Buchert R (2018) Brain perfusion imaging under Acetazolamide challenge for detection of impaired cerebrovascular reserve capacity: positive findings with (15)O-Water PET in patients with negative (99m)Tc-HMPAO SPECT findings. J Nucl Med 59:294–298. 10.2967/jnumed.117.195818 28729429 10.2967/jnumed.117.195818

[5] Alaraj A, Charbel FT, Amin-Hanjani S (2009) Peri-operative measures for treatment and prevention of cerebral vasospasm following subarachnoid hemorrhage. Neurol Res 31:651–659. 10.1179/174313209X382395 19133166 10.1179/174313209X382395

[6] Alsop DC, Detre JA, Golay X, Gunther M, Hendrikse J, Hernandez-Garcia L, Lu H, MacIntosh BJ, Parkes LM, Smits M, van Osch MJ, Wang DJ, Wong EC, Zaharchuk G (2015) Recommended implementation of arterial spin-labeled perfusion MRI for clinical applications: A consensus of the ISMRM perfusion study group and the European consortium for ASL in dementia. Magn Reson Med 73:102–116. 10.1002/mrm.25197 24715426 10.1002/mrm.25197 PMC4190138

[7] Amemiya S, Takao H, Watanabe Y, Takei N, Ueyama T, Kato S, Miyawaki S, Koizumi S, Abe O, Saito N (2022) Reliability and sensitivity to longitudinal CBF changes in Steno-Occlusive diseases: ASL versus (123) I-IMP-SPECT. J Magn Reson Imaging 55:1723–1732. 10.1002/jmri.27996 34780101 10.1002/jmri.27996

[8] Amin-Hanjani S, Du X, Mlinarevich N, Meglio G, Zhao M, Charbel FT (2005) The cut flow index: an intraoperative predictor of the success of extracranial-intracranial bypass for occlusive cerebrovascular disease. Neurosurgery 56:75–85. 10.1227/01.neu.0000143032.35416.41. discussion 75–85 15799795 10.1227/01.neu.0000143032.35416.41

[9] Arnone GD, Hage ZA, Charbel FT (2019) Single vessel double anastomosis for flow Augmentation - A novel technique for direct extracranial to intracranial bypass surgery. Oper Neurosurg (Hagerstown) 17:365–375. 10.1093/ons/opy396 10.1093/ons/opy396 30690506

[10] Ashley WW, Amin-Hanjani S, Alaraj A, Shin JH, Charbel FT (2008) Flow-assisted surgical cerebral revascularization. Neurosurg Focus 24:E20. 10.3171/FOC/2008/24/2/E20 18336091 10.3171/FOC/2008/24/2/E20

[11] Awano T, Sakatani K, Yokose N, Hoshino T, Fujiwara N, Nakamura S, Murata Y, Kano T, Katayama Y, Shikayama T, Miwa M (2010) EC-IC bypass function in Moyamoya disease and non-Moyamoya ischemic stroke evaluated by intraoperative indocyanine green fluorescence angiography. Adv Exp Med Biol 662:519–524. 10.1007/978-1-4419-1241-1_75 20204839 10.1007/978-1-4419-1241-1_75

[12] Awano T, Sakatani K, Yokose N, Kondo Y, Igarashi T, Hoshino T, Nakamura S, Fujiwara N, Murata Y, Katayama Y, Shikayama T, Miwa M (2010) Intraoperative EC-IC bypass blood flow assessment with indocyanine green angiography in Moyamoya and non-moyamoya ischemic stroke. World Neurosurg 73:668–674. 10.1016/j.wneu.2010.03.027 20934154 10.1016/j.wneu.2010.03.027

[13] Besachio DA, Ziegler JI, Duncan TD, Wanebo JS (2010) Computed tomographic angiography in evaluation of superficial Temporal to middle cerebral artery bypass. J Comput Assist Tomogr 34:437–439. 10.1097/RCT.0b013e3181cfbca2 20498550 10.1097/RCT.0b013e3181cfbca2

[14] Colby GP, Coon AL, Sciubba DM, Bydon A, Gailloud P, Tamargo RJ (2009) Intraoperative indocyanine green angiography for obliteration of a spinal dural arteriovenous fistula. J Neurosurg Spine 11:705–709. 10.3171/2009.6.SPINE09315 19951023 10.3171/2009.6.SPINE09315

[15] Esposito G, Della Pepa GM, Sabatino G, Gaudino S, Puca A, Maira G, Marchese E, Albanese A (2015) Bilateral flow changes after extracranial-intracranial bypass surgery in a complex setting of multiple brain-feeding arteries occlusion: the role of perfusion studies. Br J Neurosurg 29:723–725. 10.3109/02688697.2015.1023779 25812020 10.3109/02688697.2015.1023779

[16] Esposito G, Dias S, Burkhardt JK, Bozinov O, Regli L (2018) Role of indocyanine green videoangiography in identification of donor and recipient arteries in cerebral bypass surgery. Acta Neurochir Suppl 129:85–89. 10.1007/978-3-319-73739-3_12 30171318 10.1007/978-3-319-73739-3_12

[17] Esposito G, Kronenburg A, Fierstra J, Braun KP, Klijn CJ, van der Zwan A, Regli L (2015) STA-MCA bypass with encephalo-duro-myo-synangiosis combined with bifrontal encephalo-duro-periosteal-synangiosis as a one-staged revascularization strategy for pediatric Moyamoya vasculopathy. Childs Nerv Syst 31:765–772. 10.1007/s00381-015-2665-y 25722049 10.1007/s00381-015-2665-y

[18] Esposito G, Regli L (2018) Intraoperative tools for cerebral bypass surgery. Acta Neurochir (Wien) 160:775–778. 10.1007/s00701-017-3455-y 29322266 10.1007/s00701-017-3455-y

[19] Esposito G, Sebok M, Amin-Hanjani S, Regli L (2018) Cerebral bypass surgery: level of evidence and grade of recommendation. Acta Neurochir Suppl 129:73–77. 10.1007/978-3-319-73739-3_10 30171316 10.1007/978-3-319-73739-3_10

[20] Federau C, Christensen S, Zun Z, Park SW, Ni W, Moseley M, Zaharchuk G (2017) Cerebral blood flow, transit time, and apparent diffusion coefficient in Moyamoya disease before and after Acetazolamide. Neuroradiology 59:5–12. 10.1007/s00234-016-1766-y 27913820 10.1007/s00234-016-1766-y PMC8006793

[21] Fox BM, Dorschel KB, Lawton MT, Wanebo JE (2021) Pathophysiology of vascular stenosis and remodeling in Moyamoya disease. Front Neurol 12:661578. 10.3389/fneur.2021.661578 34539540 10.3389/fneur.2021.661578 PMC8446194

[22] Goldberg J, Vajkoczy P, Hecht N (2020) Indocyanine green videoangiography for recipient vessel stratification in superficial Temporal artery-middle cerebral artery bypass surgery. J Neurosurg 135:44–52. 10.3171/2020.5.JNS20642 32858511 10.3171/2020.5.JNS20642

[23] Gottwald LM, Toger J, Markenroth Bloch K, Peper ES, Coolen BF, Strijkers GJ, van Ooij P, Nederveen AJ (2020) High Spatiotemporal resolution 4D flow MRI of intracranial aneurysms at 7T in 10 minutes. AJNR Am J Neuroradiol 41:1201–1208. 10.3174/ajnr.A6603 32586964 10.3174/ajnr.A6603 PMC7357648

[24] Guzman R, Lee M, Achrol A, Bell-Stephens T, Kelly M, Do HM, Marks MP, Steinberg GK (2009) Clinical outcome after 450 revascularization procedures for Moyamoya disease. Clinical Article. J Neurosurg 111:927–935. 10.3171/2009.4.JNS081649 19463046 10.3171/2009.4.JNS081649

[25] Helthuis JHG, van Doormaal TPC, Amin-Hanjani S, Du X, Charbel FT, Hillen B, van der Zwan A (2020) A patient-specific cerebral blood flow model. J Biomech 98:109445. 10.1016/j.jbiomech.2019.109445 31708241 10.1016/j.jbiomech.2019.109445

[26] Kronenburg A, Esposito G, Fierstra J, Braun KP, Regli L (2014) Combined bypass technique for contemporary revascularization of unilateral MCA and bilateral frontal territories in Moyamoya vasculopathy. Acta Neurochir Suppl 119:65–70. 10.1007/978-3-319-02411-0_11 24728635 10.1007/978-3-319-02411-0_11

[27] Miller DR, Ashour R, Sullender CT, Dunn AK (2022) Continuous blood flow visualization with laser speckle contrast imaging during neurovascular surgery. Neurophotonics 9:021908. 10.1117/1.NPh.9.2.021908 35265733 10.1117/1.NPh.9.2.021908 PMC8900813

[28] Nariai T, Suzuki R, Hirakawa K, Maehara T, Ishii K, Senda M (1995) Vascular reserve in chronic cerebral ischemia measured by the Acetazolamide challenge test: comparison with positron emission tomography. AJNR Am J Neuroradiol 16:563–570 7793382 PMC8337663

[29] Park CS, Hartung G, Alaraj A, Du X, Charbel FT, Linninger AA (2020) Quantification of blood flow patterns in the cerebral arterial circulation of individual (human) subjects. Int J Numer Method Biomed Eng 36:e3288. 10.1002/cnm.3288 31742921 10.1002/cnm.3288

[30] Rajabzadeh-Oghaz H, Siddiqui AH, Asadollahi A, Kolega J, Tutino VM (2022) The association between hemodynamics and wall characteristics in human intracranial aneurysms: a review. Neurosurg Rev 45:49–61. 10.1007/s10143-021-01554-w 33913050 10.1007/s10143-021-01554-w

[31] Ren S, Wu W, Su C, Zhu Q, Schmidt M, Sun Y, Forman C, Speier P, Hong X, Lu S (2022) High-resolution compressed sensing time-of-flight MR angiography outperforms CT angiography for evaluating patients with Moyamoya disease after surgical revascularization. BMC Med Imaging 22:64. 10.1186/s12880-022-00790-w 35387607 10.1186/s12880-022-00790-w PMC8988403

[32] Scott RM, Smith ER (2009) Moyamoya disease and Moyamoya syndrome. N Engl J Med 360:1226–1237. 10.1056/NEJMra0804622 19297575 10.1056/NEJMra0804622

[33] Soize S, Bouquigny F, Kadziolka K, Portefaix C, Pierot L (2014) Value of 4D MR angiography at 3T compared with DSA for the follow-up of treated brain arteriovenous malformation. AJNR Am J Neuroradiol 35:1903–1909. 10.3174/ajnr.A3982 24904052 10.3174/ajnr.A3982 PMC7966260

[34] Tahhan N, Balanca B, Fierstra J, Waelchli T, Picart T, Dumot C, Eker O, Marinesco S, Radovanovic I, Cotton F, Berhouma M (2022) Intraoperative cerebral blood flow monitoring in neurosurgery: A review of contemporary technologies and emerging perspectives. Neurochirurgie 68:414–425. 10.1016/j.neuchi.2021.10.005 34895896 10.1016/j.neuchi.2021.10.005

[35] Tani S, Akiyama Y, Tokime T, Taki J, Ogino E, Nishida S (2011) Recipient targeting for revascularization using pulsed doppler ultrasonography for the treatment of an intracranial giant aneurysm. J Neurosurg 114:1069–1073. 10.3171/2010.2.JNS091245 20578804 10.3171/2010.2.JNS091245

[36] Togao O, Hiwatashi A, Obara M, Yamashita K, Momosaka D, Nishimura A, Arimura K, Hata N, Yoshimoto K, Iihara K, Van Cauteren M, Honda H (2018) 4D ASL-based MR angiography for visualization of distal arteries and leptomeningeal collateral vessels in Moyamoya disease: a comparison of techniques. Eur Radiol 28:4871–4881. 10.1007/s00330-018-5462-7 29737389 10.1007/s00330-018-5462-7

[37] Uchino H, Ito M, Fujima N, Kazumata K, Yamazaki K, Nakayama N, Kuroda S, Houkin K (2015) A novel application of four-dimensional magnetic resonance angiography using an arterial spin labeling technique for noninvasive diagnosis of Moyamoya disease. Clin Neurol Neurosurg 137:105–111. 10.1016/j.clineuro.2015.07.003 26185929 10.1016/j.clineuro.2015.07.003

[38] Wang M, Ma Y, Chen F, Zhou F, Zhang J, Zhang B (2022) Acceleration of pCASL-Based cerebral 4D MR angiography using compressed SENSE: A comparison with SENSE. Front Neurol 13:796271. 10.3389/fneur.2022.796271 35386411 10.3389/fneur.2022.796271 PMC8977489

[39] Wang M, Yang Y, Wang Y, Li M, Zhang J, Zhang B (2021) Vessel-selective 4D MRA based on ASL might potentially show better performance than 3D TOF MRA for treatment evaluation in patients with intra-extracranial bypass surgery: a prospective study. Eur Radiol 31:5263–5271. 10.1007/s00330-020-07503-3 33386981 10.1007/s00330-020-07503-3

[40] Wang R, Yu S, Alger JR, Zuo Z, Chen J, Wang R, An J, Wang B, Zhao J, Xue R, Wang DJ (2014) Multi-delay arterial spin labeling perfusion MRI in Moyamoya disease–comparison with CT perfusion imaging. Eur Radiol 24:1135–1144. 10.1007/s00330-014-3098-9 24557051 10.1007/s00330-014-3098-9 PMC4143230

[41] Williams DS, Detre JA, Leigh JS, Koretsky AP (1992) Magnetic resonance imaging of perfusion using spin inversion of arterial water. Proc Natl Acad Sci U S A 89:212–216. 10.1073/pnas.89.1.212 1729691 10.1073/pnas.89.1.212 PMC48206

[42] Woitzik J, Horn P, Vajkoczy P, Schmiedek P (2005) Intraoperative control of extracranial-intracranial bypass patency by near-infrared indocyanine green videoangiography. J Neurosurg 102:692–698. 10.3171/jns.2005.102.4.0692 15871512 10.3171/jns.2005.102.4.0692

[43] Yamada I, Suzuki S, Matsushima Y (1995) Moyamoya disease: diagnostic accuracy of MRI. Neuroradiology 37:356–361. 10.1007/BF00588011 7477833 10.1007/BF00588011

[44] Yim B, Gauden AJ, Steinberg GK (2022) Application of FLOW 800 in extracranial-to-intracranial bypass surgery for Moyamoya disease. Neurosurg Focus Video 6:V16. 10.3171/2021.10.FOCVID21191 36284597 10.3171/2021.10.FOCVID21191 PMC9555355

[45] Yoon S, Burkhardt JK, Lawton MT (2018) Long-term patency in cerebral revascularization surgery: an analysis of a consecutive series of 430 bypasses. J Neurosurg 131:80–87. 10.3171/2018.3.JNS172158 30141754 10.3171/2018.3.JNS172158

[46] Zhao M, Amin-Hanjani S, Ruland S, Curcio AP, Ostergren L, Charbel FT (2007) Regional cerebral blood flow using quantitative MR angiography. AJNR Am J Neuroradiol 28:1470–1473. 10.3174/ajnr.A0582 17846193 10.3174/ajnr.A0582 PMC8134363

[47] Zhao MY, Armindo RD, Gauden AJ, Yim B, Tong E, Moseley M, Steinberg GK, Zaharchuk G (2023) Revascularization improves vascular hemodynamics - a study assessing cerebrovascular reserve and transit time in Moyamoya patients using MRI. J Cereb Blood Flow Metab 43:138–151. 10.1177/0271678X221140343 36408536 10.1177/0271678X221140343 PMC10638998

[48] Zhao MY, Fan AP, Chen DY, Ishii Y, Khalighi MM, Moseley M, Steinberg GK, Zaharchuk G (2022) Using arterial spin labeling to measure cerebrovascular reactivity in Moyamoya disease: insights from simultaneous PET/MRI. J Cereb Blood Flow Metab 42:1493–1506. 10.1177/0271678X221083471 35236136 10.1177/0271678X221083471 PMC9274857

