# Supplementary material for: Virtual cut flow, an innovative noninvasive 4D ASL MRI biomarker of EIC bypass patency
Source: Neurosurg Rev. 2025 May 27;48(1):447. doi: 10.1007/s10143-025-03618-7 (PMC12116611; doi:10.1007/s10143-025-03618-7)

**Supplementary Figure 2. Statistical analysis**

Results are illustrated in blot-plotted boxes using Mann-Whitney test in Prism 10.0.2 software (Graph Pad Software Inc., San Diego, CA, USA). Groups are compared between known obliterated and patent bypasses demonstrated on DSA. All quoted p-values are two-sided, with p ≤ 0.05 (*) being considered statistically significant.

1. : The median αMCA coefficient was significantly higher in patients with a patent bypass (69.21) compared to those with an occluded bypass (11.34; p < 0.05)
2. : The median αSTA coefficient was significantly higher in patients with a patent bypass (102.74) compared to those with an occluded bypass (44.74; p < 0.05)
3. : the median VCFI was significantly higher in patients with a patent bypass (87.33%) compared to those with an occluded bypass (19.87%; p < 0.05).

αMCA: coefficient line measured in Middle Cerebral Artery, αSTA: coefficient line measured in Superficial Temporal Artery, VCFI: Virtual Cut-Flow Index


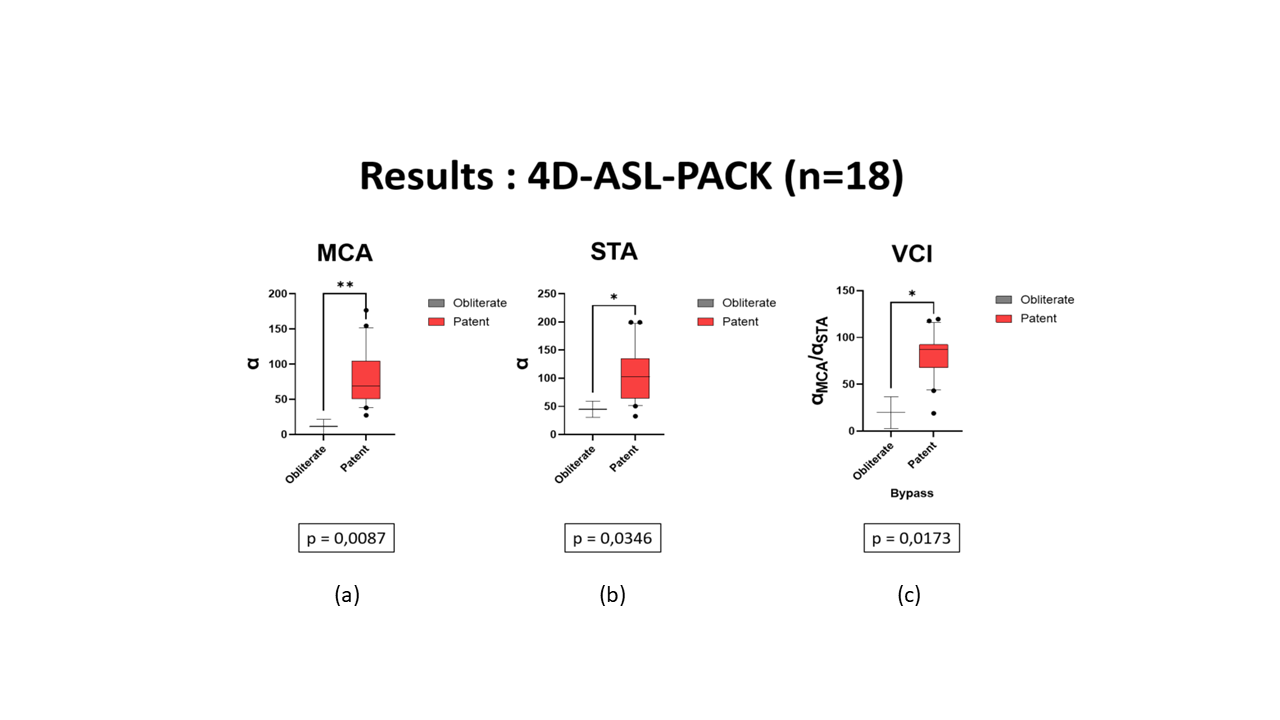

Supplement: Supplementary file 1 — Supplementary Material 1 [file 10143_2025_3618_MOESM1_ESM.docx]
